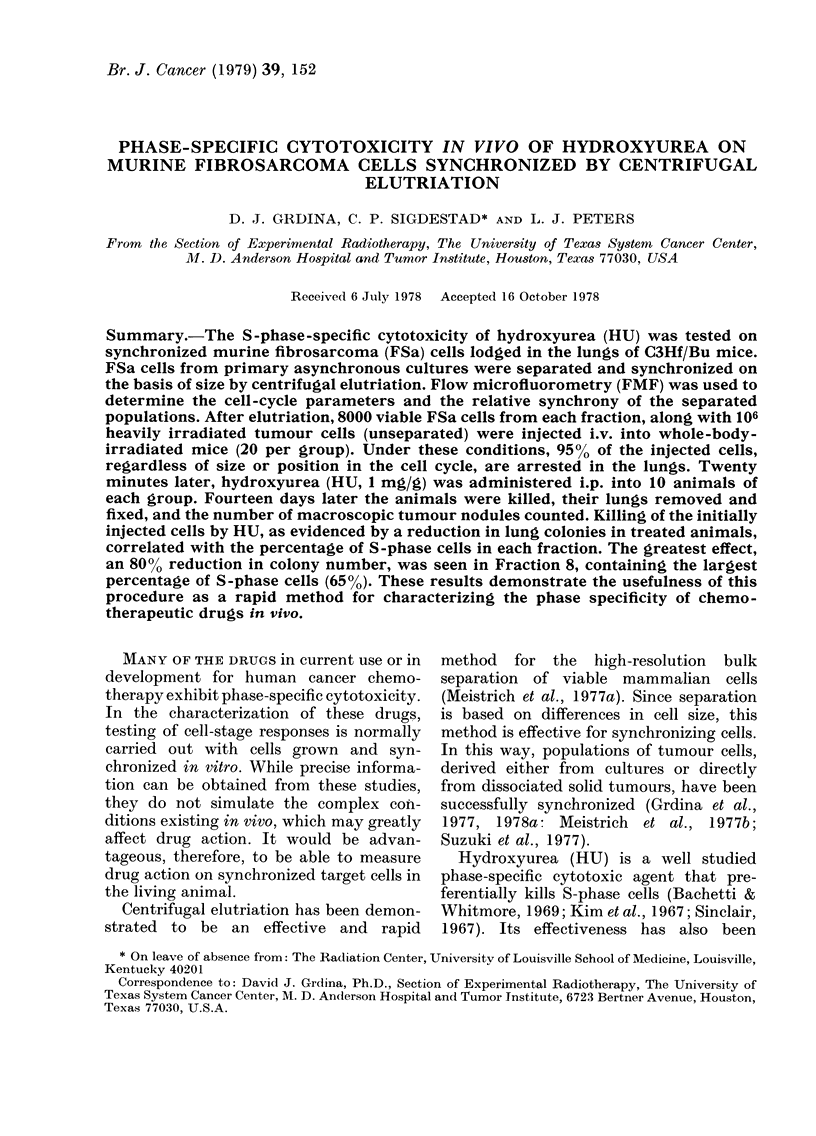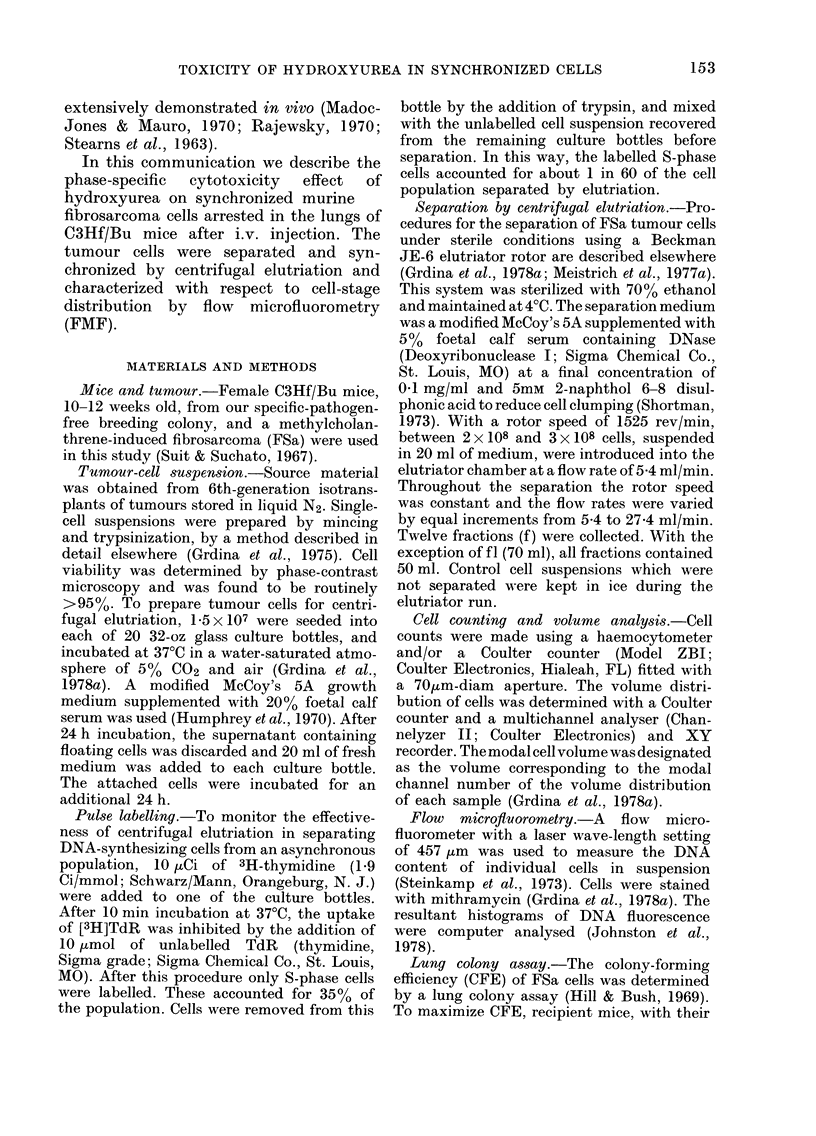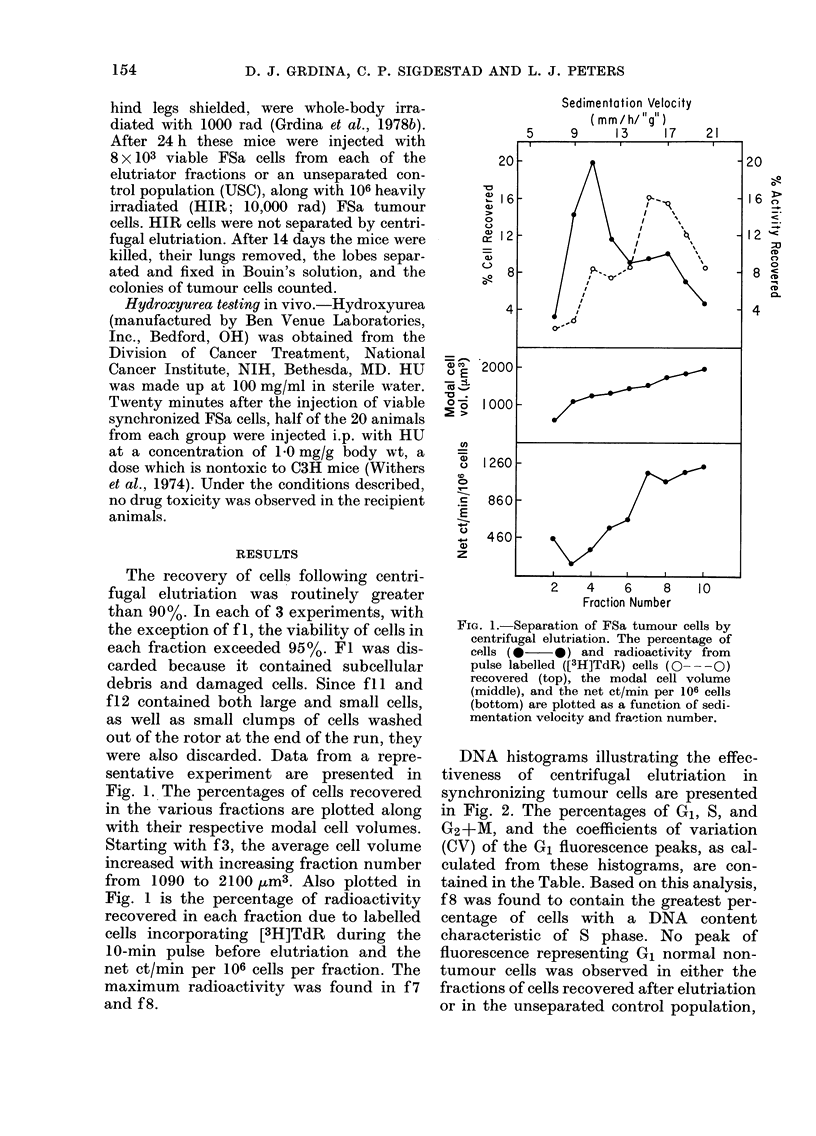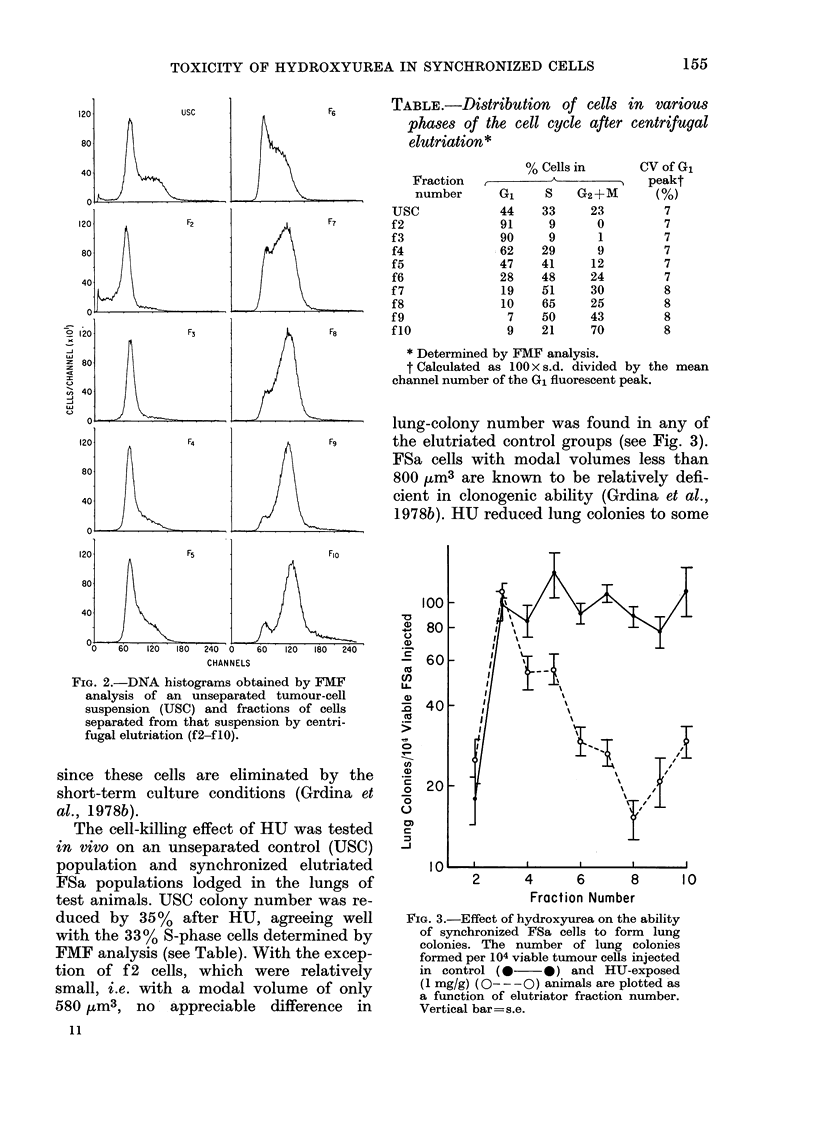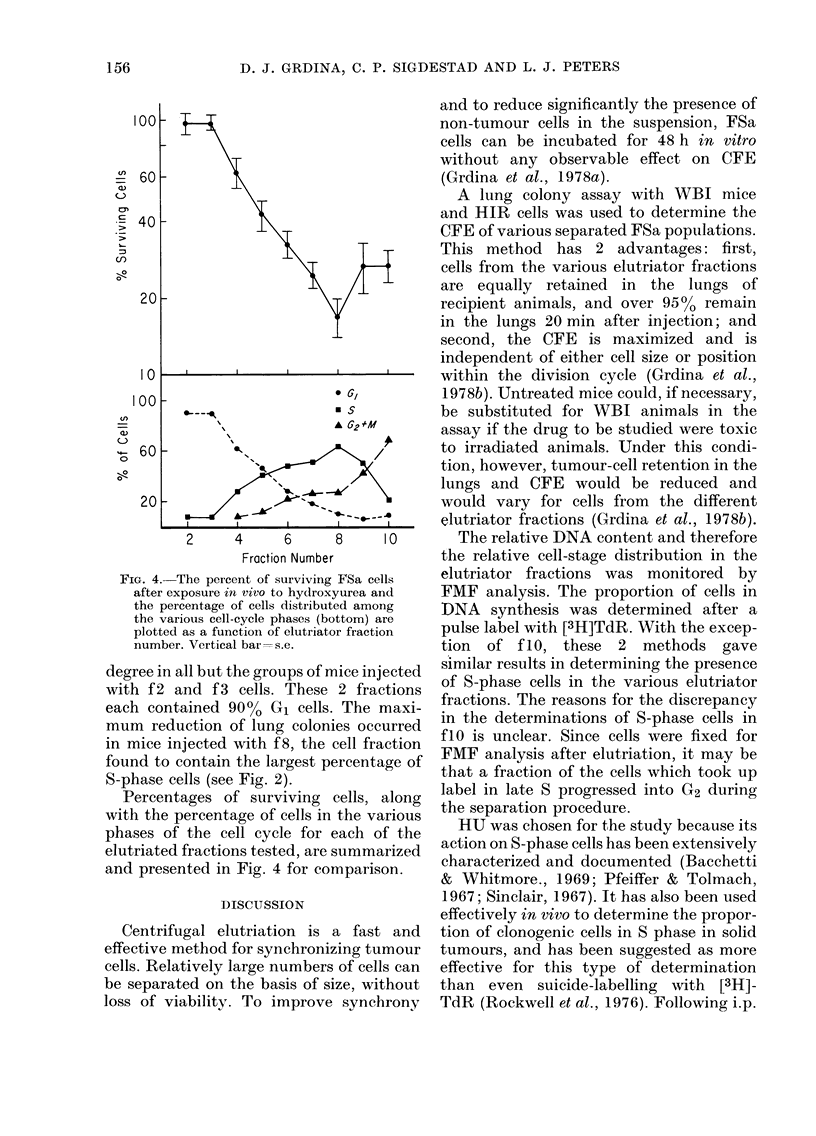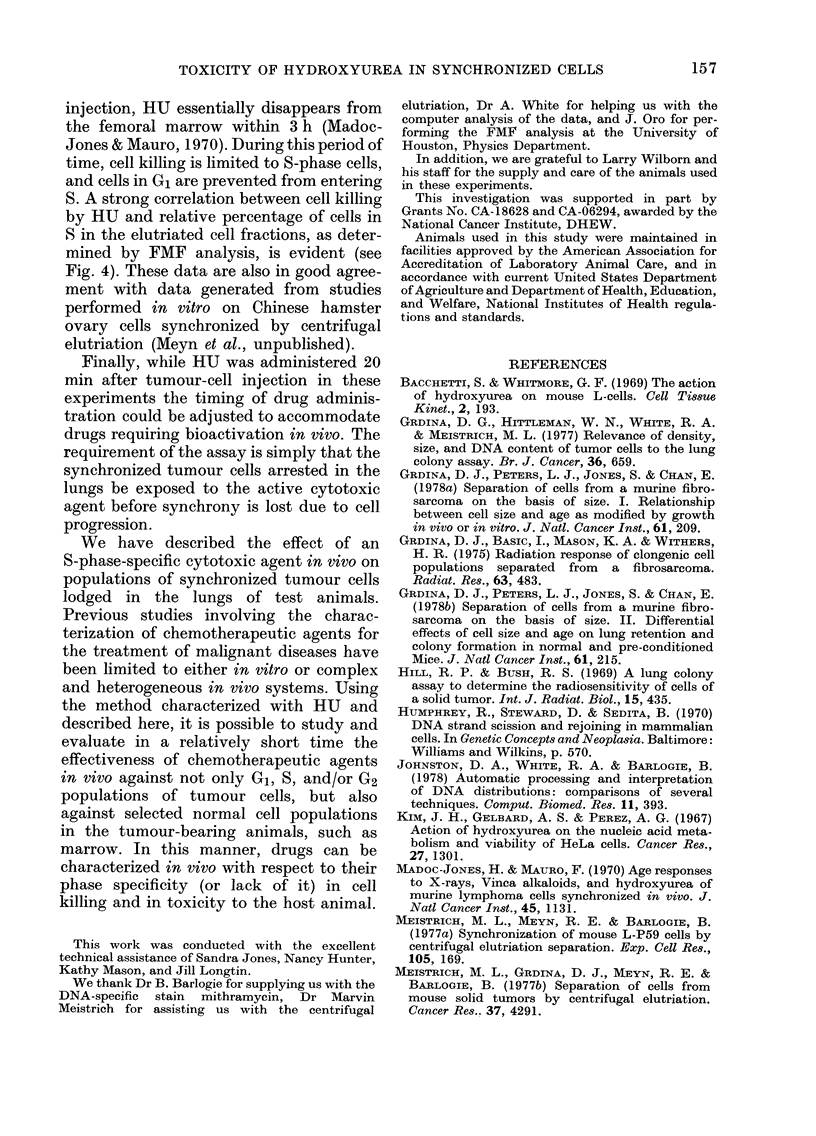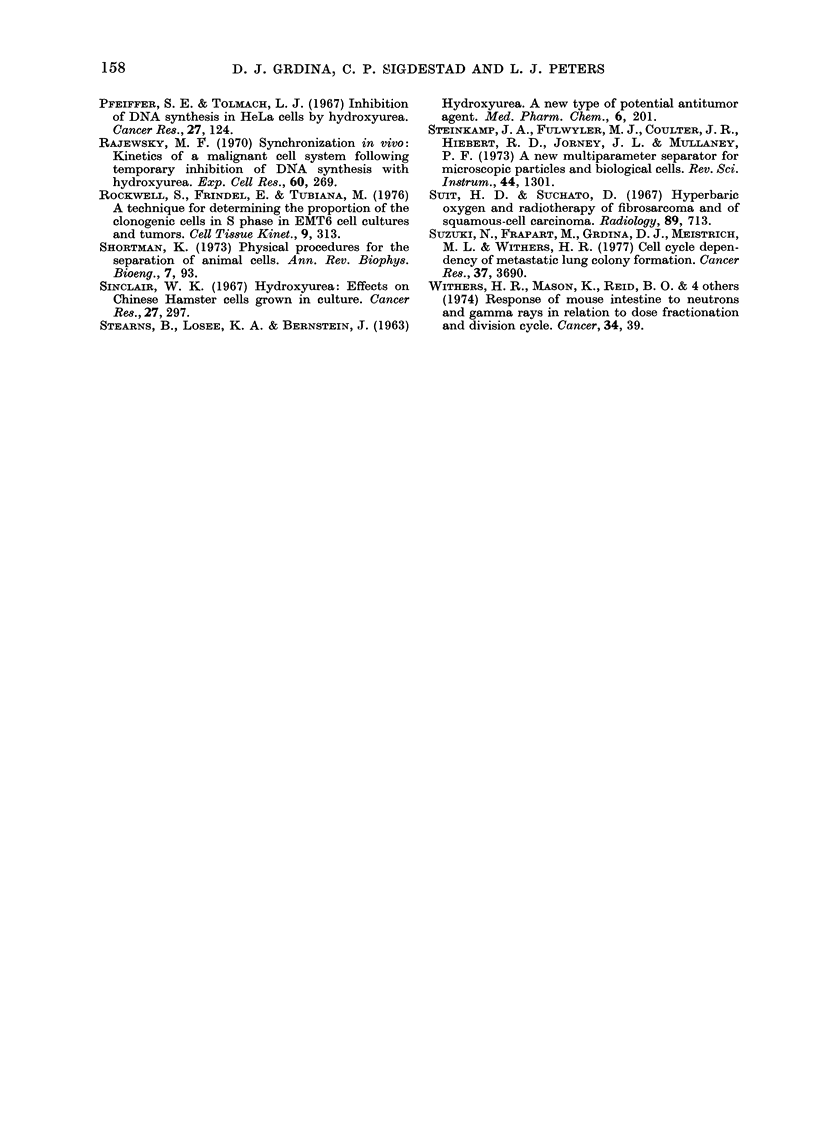# Phase-specific cytotoxicity in vivo of hydroxyurea on murine fibrosarcoma cells synchronized by centrifugal elutriation.

**DOI:** 10.1038/bjc.1979.25

**Published:** 1979-02

**Authors:** D. J. Grdina, C. P. Sigdestad, L. J. Peters

## Abstract

The S-phase-specific cytotoxicity of hydroxyurea (HU) was tested on synchronized murine fibrosarcoma (FSa) cells lodged in the lungs of C3Hf/Bu mice. FSa cells from primary asynchronous cultures were separated and synchronized on the basis of size by centrifugal elutriation. Flow microfluorometry (FMF) was used to determine the cell-cycle parameters and the relative synchrony of the separated populations. After elutriation, 8000 viable FSa cells from each fraction, along with 10(6) heavily irradiated tumour cells (unseparated) were injected i.v. into whole-body-irradiated mice (20 per group). Under these conditions, 95% of the injected cells, regardless of size or position in the cell cycle, are arrested in the lungs. Twenty minutes later, hydroxyurea (HU, 1 mg/g) was administered i.p. into 10 animals of each group. Fourteen days later the animals were killed, their lungs removed and fixed, and the number of macroscopic tumour nodules counted. Killing of the initially injected cells by HU, as evidenced by a reduction in lung colonies in treated animals, correlated with the precentage of S-phase cells in each fraction. The greatest effect, an 80% reduction in colony number, was seen in Fraction 8, containing the largest percentage of S-phase cells (65%). These results demonstrate the usefulness of this procedure as a rapid method for characterizing the phase specificity of chemotherapeutic drugs in vivo.


*Br. J. Cancer* (1979) **39**, 152

### PHASE-SPECIFIC CYTOTOXICITY *IN VIVO* OF HYDROXYUREA ON MURINE FIBROSARCOMA CELLS SYNCHRONIZED BY CENTRIFUGAL ELUTRIATION

D. J. GRDINA, C. P. SIGDESTAD* AND L. J. PETERS

*From the Section of Experimental Radiotherapy, The University of Texas System Cancer Center, M. D. Anderson Hospital and Tumor Institute, Houston, Texas* 77030, *USA*

Received 6 July 1978 Accepted 16 October 1978

**Summary.—The S-phase-specific cytotoxicity of hydroxyurea (HU) was tested on synchronized murine fibrosarcoma (FSa) cells lodged in the lungs of C3Hf/Bu mice. FSa cells from primary asynchronous cultures were separated and synchronized on the basis of size by centrifugal elutriation. Flow microfluorometry (FMF) was used to determine the cell-cycle parameters and the relative synchrony of the separated populations. After elutriation, 8000 viable FSa cells from each fraction, along with $10^6$ heavily irradiated tumour cells (unseparated) were injected i.v. into whole-body-irradiated mice (20 per group). Under these conditions, 95% of the injected cells, regardless of size or position in the cell cycle, are arrested in the lungs. Twenty minutes later, hydroxyurea (HU, 1 mg/g) was administered i.p. into 10 animals of each group. Fourteen days later the animals were killed, their lungs removed and fixed, and the number of macroscopic tumour nodules counted. Killing of the initially injected cells by HU, as evidenced by a reduction in lung colonies in treated animals, correlated with the percentage of S-phase cells in each fraction. The greatest effect, an 80% reduction in colony number, was seen in Fraction 8, containing the largest percentage of S-phase cells (65%). These results demonstrate the usefulness of this procedure as a rapid method for characterizing the phase specificity of chemotherapeutic drugs *in vivo*.**

MANY OF THE DRUGS in current use or in development for human cancer chemotherapy exhibit phase-specific cytotoxicity. In the characterization of these drugs, testing of cell-stage responses is normally carried out with cells grown and synchronized *in vitro*. While precise information can be obtained from these studies, they do not simulate the complex conditions existing *in vivo*, which may greatly affect drug action. It would be advantageous, therefore, to be able to measure drug action on synchronized target cells in the living animal.

Centrifugal elutriation has been demonstrated to be an effective and rapid method for the high-resolution bulk separation of viable mammalian cells (Meistrich *et al.*, 1977*a*). Since separation is based on differences in cell size, this method is effective for synchronizing cells. In this way, populations of tumour cells, derived either from cultures or directly from dissociated solid tumours, have been successfully synchronized (Grdina *et al.*, 1977, 1978*a*: Meistrich *et al.*, 1977*b*; Suzuki *et al.*, 1977).

Hydroxyurea (HU) is a well studied phase-specific cytotoxic agent that preferentially kills S-phase cells (Bachetti & Whitmore, 1969; Kim *et al.*, 1967; Sinclair, 1967). Its effectiveness has also been

* On leave of absence from: The Radiation Center, University of Louisville School of Medicine, Louisville, Kentucky 40201

Correspondence to: David J. Grdina, Ph.D., Section of Experimental Radiotherapy, The University of Texas System Cancer Center, M. D. Anderson Hospital and Tumor Institute, 6723 Bertner Avenue, Houston, Texas 77030, U.S.A.

extensively demonstrated *in vivo* (Madoc-Jones & Mauro, 1970; Rajewsky, 1970; Stearns *et al.*, 1963).

In this communication we describe the phase-specific cytotoxicity effect of hydroxyurea on synchronized murine fibrosarcoma cells arrested in the lungs of C3Hf/Bu mice after i.v. injection. The tumour cells were separated and synchronized by centrifugal elutriation and characterized with respect to cell-stage distribution by flow microfluorometry (FMF).

### MATERIALS AND METHODS

*Mice and tumour.*—Female C3Hf/Bu mice, 10–12 weeks old, from our specific-pathogen-free breeding colony, and a methylcholanthrene-induced fibrosarcoma (FSa) were used in this study (Suit & Suchato, 1967).

*Tumour-cell suspension.*—Source material was obtained from 6th-generation isotransplants of tumours stored in liquid $N_2$. Single-cell suspensions were prepared by mincing and trypsinization, by a method described in detail elsewhere (Grdina *et al.*, 1975). Cell viability was determined by phase-contrast microscopy and was found to be routinely >95%. To prepare tumour cells for centrifugal elutriation, $1\cdot5 \times 10^7$ were seeded into each of 20 32-oz glass culture bottles, and incubated at 37°C in a water-saturated atmosphere of 5% $CO_2$ and air (Grdina *et al.*, 1978*a*). A modified McCoy's 5A growth medium supplemented with 20% foetal calf serum was used (Humphrey *et al.*, 1970). After 24 h incubation, the supernatant containing floating cells was discarded and 20 ml of fresh medium was added to each culture bottle. The attached cells were incubated for an additional 24 h.

*Pulse labelling.*—To monitor the effectiveness of centrifugal elutriation in separating DNA-synthesizing cells from an asynchronous population, 10 $\mu$Ci of $^3$H-thymidine (1·9 Ci/mmol; Schwarz/Mann, Orangeburg, N. J.) were added to one of the culture bottles. After 10 min incubation at 37°C, the uptake of [$^3$H]TdR was inhibited by the addition of 10 $\mu$mol of unlabelled TdR (thymidine, Sigma grade; Sigma Chemical Co., St. Louis, MO). After this procedure only S-phase cells were labelled. These accounted for 35% of the population. Cells were removed from this bottle by the addition of trypsin, and mixed with the unlabelled cell suspension recovered from the remaining culture bottles before separation. In this way, the labelled S-phase cells accounted for about 1 in 60 of the cell population separated by elutriation.

*Separation by centrifugal elutriation.*—Procedures for the separation of FSa tumour cells under sterile conditions using a Beckman JE-6 elutriator rotor are described elsewhere (Grdina *et al.*, 1978*a*; Meistrich *et al.*, 1977*a*). This system was sterilized with 70% ethanol and maintained at 4°C. The separation medium was a modified McCoy's 5A supplemented with 5% foetal calf serum containing DNase (Deoxyribonuclease I; Sigma Chemical Co., St. Louis, MO) at a final concentration of 0·1 mg/ml and 5mM 2-naphthol 6–8 disulphonic acid to reduce cell clumping (Shortman, 1973). With a rotor speed of 1525 rev/min, between $2 \times 10^8$ and $3 \times 10^8$ cells, suspended in 20 ml of medium, were introduced into the elutriator chamber at a flow rate of 5·4 ml/min. Throughout the separation the rotor speed was constant and the flow rates were varied by equal increments from 5·4 to 27·4 ml/min. Twelve fractions (f) were collected. With the exception of f1 (70 ml), all fractions contained 50 ml. Control cell suspensions which were not separated were kept in ice during the elutriator run.

*Cell counting and volume analysis.*—Cell counts were made using a haemocytometer and/or a Coulter counter (Model ZBI; Coulter Electronics, Hialeah, FL) fitted with a 70$\mu$m-diam aperture. The volume distribution of cells was determined with a Coulter counter and a multichannel analyser (Channelyzer II; Coulter Electronics) and XY recorder. The modal cell volume was designated as the volume corresponding to the modal channel number of the volume distribution of each sample (Grdina *et al.*, 1978*a*).

*Flow microfluorometry.*—A flow microfluorometer with a laser wave-length setting of 457 $\mu$m was used to measure the DNA content of individual cells in suspension (Steinkamp *et al.*, 1973). Cells were stained with mithramycin (Grdina *et al.*, 1978*a*). The resultant histograms of DNA fluorescence were computer analysed (Johnston *et al.*, 1978).

*Lung colony assay.*—The colony-forming efficiency (CFE) of FSa cells was determined by a lung colony assay (Hill & Bush, 1969). To maximize CFE, recipient mice, with their hind legs shielded, were whole-body irradiated with 1000 rad (Grdina *et al.*, 1978*b*). After 24 h these mice were injected with $8 \times 10^3$ viable FSa cells from each of the elutriator fractions or an unseparated control population (USC), along with $10^6$ heavily irradiated (HIR; 10,000 rad) FSa tumour cells. HIR cells were not separated by centrifugal elutriation. After 14 days the mice were killed, their lungs removed, the lobes separated and fixed in Bouin's solution, and the colonies of tumour cells counted.

*Hydroxyurea testing* in vivo.—Hydroxyurea (manufactured by Ben Venue Laboratories, Inc., Bedford, OH) was obtained from the Division of Cancer Treatment, National Cancer Institute, NIH, Bethesda, MD. HU was made up at 100 mg/ml in sterile water. Twenty minutes after the injection of viable synchronized FSa cells, half of the 20 animals from each group were injected i.p. with HU at a concentration of 1·0 mg/g body wt, a dose which is nontoxic to C3H mice (Withers *et al.*, 1974). Under the conditions described, no drug toxicity was observed in the recipient animals.

### RESULTS

The recovery of cells following centrifugal elutriation was routinely greater than 90%. In each of 3 experiments, with the exception of f1, the viability of cells in each fraction exceeded 95%. F1 was discarded because it contained subcellular debris and damaged cells. Since f11 and f12 contained both large and small cells, as well as small clumps of cells washed out of the rotor at the end of the run, they were also discarded. Data from a representative experiment are presented in Fig. 1. The percentages of cells recovered in the various fractions are plotted along with their respective modal cell volumes. Starting with f3, the average cell volume increased with increasing fraction number from 1090 to 2100 $\mu m^3$. Also plotted in Fig. 1 is the percentage of radioactivity recovered in each fraction due to labelled cells incorporating [$^3$H]TdR during the 10-min pulse before elutriation and the net ct/min per $10^6$ cells per fraction. The maximum radioactivity was found in f7 and f8.

FIG. 1.—Separation of FSa tumour cells by centrifugal elutriation. The percentage of cells (●——●) and radioactivity from pulse labelled ([$^3$H]TdR) cells (○---○) recovered (top), the modal cell volume (middle), and the net ct/min per $10^6$ cells (bottom) are plotted as a function of sedimentation velocity and fraction number.

DNA histograms illustrating the effectiveness of centrifugal elutriation in synchronizing tumour cells are presented in Fig. 2. The percentages of $G_1$, S, and $G_2$+M, and the coefficients of variation (CV) of the $G_1$ fluorescence peaks, as calculated from these histograms, are contained in the Table. Based on this analysis, f8 was found to contain the greatest percentage of cells with a DNA content characteristic of S phase. No peak of fluorescence representing $G_1$ normal non-tumour cells was observed in either the fractions of cells recovered after elutriation or in the unseparated control population,

FIG. 2.—DNA histograms obtained by FMF analysis of an unseparated tumour-cell suspension (USC) and fractions of cells separated from that suspension by centrifugal elutriation (f2–f10).

since these cells are eliminated by the short-term culture conditions (Grdina *et al.*, 1978*b*).

The cell-killing effect of HU was tested *in vivo* on an unseparated control (USC) population and synchronized elutriated FSa populations lodged in the lungs of test animals. USC colony number was reduced by 35% after HU, agreeing well with the 33% S-phase cells determined by FMF analysis (see Table). With the exception of f2 cells, which were relatively small, *i.e.* with a modal volume of only 580 $\mu m^3$, no appreciable difference in

TABLE.—*Distribution of cells in various phases of the cell cycle after centrifugal elutriation**

| Fraction number | % Cells in $G_1$ | S | $G_2+M$ | CV of $G_1$ peak† (%) |
|---|---|---|---|---|
| USC | 44 | 33 | 23 | 7 |
| f2 | 91 | 9 | 0 | 7 |
| f3 | 90 | 9 | 1 | 7 |
| f4 | 62 | 29 | 9 | 7 |
| f5 | 47 | 41 | 12 | 7 |
| f6 | 28 | 48 | 24 | 7 |
| f7 | 19 | 51 | 30 | 8 |
| f8 | 10 | 65 | 25 | 8 |
| f9 | 7 | 50 | 43 | 8 |
| f10 | 9 | 21 | 70 | 8 |

* Determined by FMF analysis.
† Calculated as 100×s.d. divided by the mean channel number of the $G_1$ fluorescent peak.

lung-colony number was found in any of the elutriated control groups (see Fig. 3). FSa cells with modal volumes less than 800 $\mu m^3$ are known to be relatively deficient in clonogenic ability (Grdina *et al.*, 1978*b*). HU reduced lung colonies to some

FIG. 3.—Effect of hydroxyurea on the ability of synchronized FSa cells to form lung colonies. The number of lung colonies formed per $10^4$ viable tumour cells injected in control (●——●) and HU-exposed (1 mg/g) (○– – –○) animals are plotted as a function of elutriator fraction number. Vertical bar = s.e.

FIG. 4.—The percent of surviving FSa cells after exposure *in vivo* to hydroxyurea and the percentage of cells distributed among the various cell-cycle phases (bottom) are plotted as a function of elutriator fraction number. Vertical bar = s.e.

degree in all but the groups of mice injected with f2 and f3 cells. These 2 fractions each contained 90% $G_1$ cells. The maximum reduction of lung colonies occurred in mice injected with f8, the cell fraction found to contain the largest percentage of S-phase cells (see Fig. 2).

Percentages of surviving cells, along with the percentage of cells in the various phases of the cell cycle for each of the elutriated fractions tested, are summarized and presented in Fig. 4 for comparison.

### DISCUSSION

Centrifugal elutriation is a fast and effective method for synchronizing tumour cells. Relatively large numbers of cells can be separated on the basis of size, without loss of viability. To improve synchrony and to reduce significantly the presence of non-tumour cells in the suspension, FSa cells can be incubated for 48 h *in vitro* without any observable effect on CFE (Grdina *et al.*, 1978*a*).

A lung colony assay with WBI mice and HIR cells was used to determine the CFE of various separated FSa populations. This method has 2 advantages: first, cells from the various elutriator fractions are equally retained in the lungs of recipient animals, and over 95% remain in the lungs 20 min after injection; and second, the CFE is maximized and is independent of either cell size or position within the division cycle (Grdina *et al.*, 1978*b*). Untreated mice could, if necessary, be substituted for WBI animals in the assay if the drug to be studied were toxic to irradiated animals. Under this condition, however, tumour-cell retention in the lungs and CFE would be reduced and would vary for cells from the different elutriator fractions (Grdina *et al.*, 1978*b*).

The relative DNA content and therefore the relative cell-stage distribution in the elutriator fractions was monitored by FMF analysis. The proportion of cells in DNA synthesis was determined after a pulse label with [$^3$H]TdR. With the exception of f10, these 2 methods gave similar results in determining the presence of S-phase cells in the various elutriator fractions. The reasons for the discrepancy in the determinations of S-phase cells in f10 is unclear. Since cells were fixed for FMF analysis after elutriation, it may be that a fraction of the cells which took up label in late S progressed into $G_2$ during the separation procedure.

HU was chosen for the study because its action on S-phase cells has been extensively characterized and documented (Bacchetti & Whitmore., 1969; Pfeiffer & Tolmach, 1967; Sinclair, 1967). It has also been used effectively *in vivo* to determine the proportion of clonogenic cells in S phase in solid tumours, and has been suggested as more effective for this type of determination than even suicide-labelling with [$^3$H]-TdR (Rockwell *et al.*, 1976). Following i.p.

injection, HU essentially disappears from the femoral marrow within 3 h (Madoc-Jones & Mauro, 1970). During this period of time, cell killing is limited to S-phase cells, and cells in $G_1$ are prevented from entering S. A strong correlation between cell killing by HU and relative percentage of cells in S in the elutriated cell fractions, as determined by FMF analysis, is evident (see Fig. 4). These data are also in good agreement with data generated from studies performed *in vitro* on Chinese hamster ovary cells synchronized by centrifugal elutriation (Meyn *et al.*, unpublished).

Finally, while HU was administered 20 min after tumour-cell injection in these experiments the timing of drug administration could be adjusted to accommodate drugs requiring bioactivation *in vivo*. The requirement of the assay is simply that the synchronized tumour cells arrested in the lungs be exposed to the active cytotoxic agent before synchrony is lost due to cell progression.

We have described the effect of an S-phase-specific cytotoxic agent *in vivo* on populations of synchronized tumour cells lodged in the lungs of test animals. Previous studies involving the characterization of chemotherapeutic agents for the treatment of malignant diseases have been limited to either *in vitro* or complex and heterogeneous *in vivo* systems. Using the method characterized with HU and described here, it is possible to study and evaluate in a relatively short time the effectiveness of chemotherapeutic agents *in vivo* against not only $G_1$, S, and/or $G_2$ populations of tumour cells, but also against selected normal cell populations in the tumour-bearing animals, such as marrow. In this manner, drugs can be characterized *in vivo* with respect to their phase specificity (or lack of it) in cell killing and in toxicity to the host animal.

This work was conducted with the excellent technical assistance of Sandra Jones, Nancy Hunter, Kathy Mason, and Jill Longtin.

We thank Dr B. Barlogie for supplying us with the DNA-specific stain mithramycin, Dr Marvin Meistrich for assisting us with the centrifugal elutriation, Dr A. White for helping us with the computer analysis of the data, and J. Oro for performing the FMF analysis at the University of Houston, Physics Department.

In addition, we are grateful to Larry Wilborn and his staff for the supply and care of the animals used in these experiments.

This investigation was supported in part by Grants No. CA-18628 and CA-06294, awarded by the National Cancer Institute, DHEW.

Animals used in this study were maintained in facilities approved by the American Association for Accreditation of Laboratory Animal Care, and in accordance with current United States Department of Agriculture and Department of Health, Education, and Welfare, National Institutes of Health regulations and standards.

### REFERENCES

BACCHETTI, S. & WHITMORE, G. F. (1969) The action of hydroxyurea on mouse L-cells. *Cell Tissue Kinet.*, **2**, 193.

GRDINA, D. G., HITTLEMAN, W. N., WHITE, R. A. & MEISTRICH, M. L. (1977) Relevance of density, size, and DNA content of tumor cells to the lung colony assay. *Br. J. Cancer*, **36**, 659.

GRDINA, D. J., PETERS, L. J., JONES, S. & CHAN, E. (1978*a*) Separation of cells from a murine fibrosarcoma on the basis of size. I. Relationship between cell size and age as modified by growth *in vivo* or *in vitro*. *J. Natl. Cancer Inst.*, **61**, 209.

GRDINA, D. J., BASIC, I., MASON, K. A. & WITHERS, H. R. (1975) Radiation response of clongenic cell populations separated from a fibrosarcoma. *Radiat. Res.*, **63**, 483.

GRDINA, D. J., PETERS, L. J., JONES, S. & CHAN, E. (1978*b*) Separation of cells from a murine fibrosarcoma on the basis of size. II. Differential effects of cell size and age on lung retention and colony formation in normal and pre-conditioned Mice. *J. Natl Cancer Inst.*, **61**, 215.

HILL, R. P. & BUSH, R. S. (1969) A lung colony assay to determine the radiosensitivity of cells of a solid tumor. *Int. J. Radiat. Biol.*, **15**, 435.

HUMPHREY, R., STEWARD, D. & SEDITA, B. (1970) DNA strand scission and rejoining in mammalian cells. In *Genetic Concepts and Neoplasia*. Baltimore: Williams and Wilkins, p. 570.

JOHNSTON, D. A., WHITE, R. A. & BARLOGIE, B. (1978) Automatic processing and interpretation of DNA distributions: comparisons of several techniques. *Comput. Biomed. Res.* **11**, 393.

KIM, J. H., GELBARD, A. S. & PEREZ, A. G. (1967) Action of hydroxyurea on the nucleic acid metabolism and viability of HeLa cells. *Cancer Res.*, **27**, 1301.

MADOC-JONES, H. & MAURO, F. (1970) Age responses to X-rays, Vinca alkaloids, and hydroxyurea of murine lymphoma cells synchronized *in vivo*. *J. Natl Cancer Inst.*, **45**, 1131.

MEISTRICH, M. L., MEYN, R. E. & BARLOGIE, B. (1977*a*) Synchronization of mouse L-P59 cells by centrifugal elutriation separation. *Exp. Cell Res.*, **105**, 169.

MEISTRICH, M. L., GRDINA, D. J., MEYN, R. E. & BARLOGIE, B. (1977*b*) Separation of cells from mouse solid tumors by centrifugal elutriation. *Cancer Res.*. **37**, 4291.

PFEIFFER, S. E. & TOLMACH, L. J. (1967) Inhibition of DNA synthesis in HeLa cells by hydroxyurea. *Cancer Res.*, **27**, 124.

RAJEWSKY, M. F. (1970) Synchronization *in vivo*: Kinetics of a malignant cell system following temporary inhibition of DNA synthesis with hydroxyurea. *Exp. Cell Res.*, **60**, 269.

ROCKWELL, S., FRINDEL, E. & TUBIANA, M. (1976) A technique for determining the proportion of the clonogenic cells in S phase in EMT6 cell cultures and tumors. *Cell Tissue Kinet.*, **9**, 313.

SHORTMAN, K. (1973) Physical procedures for the separation of animal cells. *Ann. Rev. Biophys. Bioeng.*, **7**, 93.

SINCLAIR, W. K. (1967) Hydroxyurea: Effects on Chinese Hamster cells grown in culture. *Cancer Res.*, **27**, 297.

STEARNS, B., LOSEE, K. A. & BERNSTEIN, J. (1963) Hydroxyurea. A new type of potential antitumor agent. *Med. Pharm. Chem.*, **6**, 201.

STEINKAMP, J. A., FULWYLER, M. J., COULTER, J. R., HIEBERT, R. D., JORNEY, J. L. & MULLANEY, P. F. (1973) A new multiparameter separator for microscopic particles and biological cells. *Rev. Sci. Instrum.*, **44**, 1301.

SUIT, H. D. & SUCHATO, D. (1967) Hyperbaric oxygen and radiotherapy of fibrosarcoma and of squamous-cell carcinoma. *Radiology*, **89**, 713.

SUZUKI, N., FRAPART, M., GRDINA, D. J., MEISTRICH, M. L. & WITHERS, H. R. (1977) Cell cycle dependency of metastatic lung colony formation. *Cancer Res.*, **37**, 3690.

WITHERS, H. R., MASON, K., REID, B. O. & 4 others (1974) Response of mouse intestine to neutrons and gamma rays in relation to dose fractionation and division cycle. *Cancer*, **34**, 39.